# Risk factors for oxygen requirement in hospitalized pregnant and postpartum women with COVID-19

**DOI:** 10.1016/j.clinsp.2022.100072

**Published:** 2022-06-20

**Authors:** Fernanda Spadotto Baptista, Cristiane Freitas Paganoti, Ursula Trovato Gomez, Stela Verzinhasse Peres, Luiz Marcelo Malbouisson, Maria de Lourdes Brizot, Rossana Pulcineli Vieira Francisco

**Affiliations:** a Disciplina de Obstetrícia, Departamento de Obstetrícia e Ginecologia, Faculdade de Medicina, Universidade de São Paulo (FMUSP), São Paulo, SP, Brazil; b Divisão de Clínica Obstétrica, Hospital das Clínicas, Faculdade de Medicina, Universidade de São Paulo (HCFMUSP), São Paulo, SP, Brazil; c Disciplina de Anestesiologia, Departamento de Cirurgia, Faculdade de Medicina, Universidade de São Paulo (FMUSP), São Paulo, SP, Brazil

**Keywords:** COVID-19, Risk factors, Pregnancy, Maternal mortality, Oxygen supply, Intensive care unit, Severe acute respiratory syndrome

## Abstract

**Objective:**

To identify risk factors for Oxygen (O_2_) needs in pregnant and postpartum women with COVID-19.

**Methods:**

Prospective cohort involving pregnant women hospitalized with COVID-19 from April to October 2020. The oxygen need was analyzed regarding risk factors: demographic characteristics, clinical and laboratory parameters at hospital admission, and chest Computer Tomography (CT) findings. Poisson univariate analysis was used to estimate the Relative Risk (RR) and 95% Confidence Intervals.

**Results:**

145 patients, 80 who used and 65 who did not use O_2_, were included. Body mass index ≥ 30, smoking, and chronic hypertension increased the risk of O_2_ need by 1.86 (95% CI 1.10–3.21), 1.57 (95% CI 1.16‒2.12), and 1.46 (95% CI 1.09‒1.95), respectively. Patients who were hospitalized for COVID-19 and for obstetric reasons had 8.24 (95% CI 2.8‒24.29) and 3.44 (95% CI 1.05‒11.31) times more use of O_2_ than those admitted for childbirth and abortion. Respiratory rate ≥ 24 breaths/min and O_2_ saturation < 95% presented RR for O_2_ requirements of 2.55 (1.82‒3.56) and 1.68 (95% CI 1.27–2.20), respectively. Ground Glass (GG) < 50% and with GG ≥ 50%, the risk of O_2_ use were respectively 3.41-fold and 5.33-fold higher than in patients who haven't viral pneumonia on CT. The combination of C-reactive protein ≥ 21 mg/L, hemoglobin < 11.0 g/dL, and lymphopenia < 1500 mm^3^ on hospital admission increased the risk of O_2_ use by 4.97-times.

**Conclusions:**

In obstetric patients, clinical history, laboratory, clinical and radiological parameters at admission were identified as a risk for O_2_ need, selecting the population with the greatest chance of worsening.

## Highlights

• In unvaccinated pregnant and postpartum women, any need for oxygen supply increases the risk of invasive ventilation.
• Obesity, smoking and chronic arterial hypertension proved to be risk factors for the use of oxygen in pregnant and postpartum women with COVID-19.
• The combination of C-reactive protein ≥ 21 mg/L, hemoglobin < 11.0 g/dL, and lymphopenia < 1500 mm^3^ on hospital admission and the presence of ground glass ≥ 50% in computer tomography increased the risk of O_2_ use by 4.97 and 5.33 times respectively in pregnant and postpartum women with COVID-19.

## Introduction

Since the World Health Organization declared the new SARS-CoV-2 pandemic installed in March 2020, an avalanche of knowledge and discoveries has hit us. Many protocol changes have occurred, including the identification of pregnant women as a risk group for progression to severe forms of the disease and, therefore, at greater risk of needing oxygen support and Orotracheal Intubation (OTI).^1^, ^2^, ^3^

Developing countries, which already had difficulties in reducing maternal death and near-miss rates, quickly faced an increase in maternal death from COVID-19. In this context, Brazil has surpassed 1,800 cases of maternal death due to COVID during the pandemic.^4^ This increase in maternal mortality has been pointed out by studies that reinforce socioeconomic inequalities and the difficulty in structuring the health system to care for severe cases of diseases in pregnant and postpartum women.^4^, ^5^, ^6^

The identification of patients at greater risk of clinical deterioration has been investigated, especially in the general population.^7^ Several studies propose risk factors for admission to the Intensive Care Units (ICU), orotracheal intubation, and death.^8^, ^9^, ^10^ Regarding the pathophysiology of COVID-19, it is known that the need to use O_2_ can be considered a sentinel event since from this evolution there is a risk of worsening the respiratory condition, often quickly.^11^, ^12^

Being able to screen pregnant women at higher risk of O_2_, use would prioritize care for the maternal-fetal binomial and, mainly, greater access to ICU and OTI. Thus, considering that the clinical deterioration of the disease most often implies the onset of severe acute respiratory syndrome, requiring oxygen support,^11^^,^^12^ this study aims to identify the risk factors for the need for oxygen during hospitalization of pregnant postpartum women with COVID-19.

## Materials and methods

The data analyzed in this study are part of the cohort study “Exploratory study on COVID-19 in pregnancy” Data were selected concerning pregnant and postpartum women hospitalized at *Hospital das Clínicas da Faculdade de Medicina da Universidade de São Paulo* with COVID-19 (with flu-like symptoms or severe acute respiratory syndrome) confirmed by positive laboratory SARS-CoV-2 test, from April to October 2020.

The use of O_2_ during pregnancy was indicated to ensure the following clinical parameters: O_2_ saturation greater than or equal to 95% (if postpartum, greater than or equal to 92%), a respiratory rate between 20 and 24, the avoidance of hypercapnia (pCO_2_ > 45 mmHg) during assisted ventilation, correction and treatment of respiratory effort and the support of cardiovascular stability. To ensure these parameters, the supply of O_2_ occurs progressively, and when the maximum supply of each device is reached, it passes on to the next. It starts with a nasal catheter (gradually increasing to a maximum flow of 6 liters/minute) and progresses respectively to a face mask (maximum of 15 liters/minute with FiO_2_ at 50%), a high-flow nasal cannula (maximum 40 to 70 liters/minute), non-invasive ventilation, and finally orotracheal intubation.^11^^,^^12^

Indications for admission to the intensive care unit included: O_2_ saturation < 95% despite O_2_ catheter at 6 liters/minute, ventilatory effort despite O_2_ supply, PaO_2_/FiO_2_ ratio (partial pressure of arterial O_2_/inspired O_2_ fraction) < 300, arterial hypotension (mean arterial pressure < 65 mmHg), altered peripheral perfusion, altered level of consciousness and renal dysfunction.^11^^,^^12^

For the analysis, two groups were compared, one with O_2_ need and the other without O_2_ concerning the following factors:

• Demographic: maternal age, body mass index at admission, smoking.
• Clinical: blood type (divided into type O and not O),^13^ pre-existing maternal comorbidities (chronic arterial hypertension, pneumopathy, cardiopathy, diabetes, rheumatologic diseases, and neurological diseases).
• Obstetric history: pregnant woman, postpartum woman, presence of pre-eclampsia and/or gestational diabetes in this pregnancy.
• Reason for hospitalization: admission due to delivery or abortion, when the patient was admitted to labor and delivery or abortion but had mild symptoms of COVID; hospitalization due to COVID-19, when symptoms of COVID-19 indicated hospitalization; and admission for other reasons, which included patients who were diagnosed with COVID-19 and hospitalized for reasons related to pregnancy (premature rupture of membranes, preterm labor, diabetes, etc.).
• Factors related to COVID-19: gestational age at onset of symptoms, days since onset of symptoms at hospital admission, types of symptoms (were considered a fever, cough, odynophagia, myalgia, asthenia, runny nose, diarrhea, anosmia, dysgeusia, dyspnea, headache, and fatigue).
• Clinical parameters on admission: heart rate, respiratory rate, blood pressure, body temperature, and oxygen saturation.
• Laboratory parameters on admission: hemoglobin, leukocytes, lymphocytes, neutrophils, neutrophil/lymphocyte ratio, platelets, C-Reactive Protein (CPR), aspartate aminotransferase, alanine aminotransferase, lactic dehydrogenase, creatine phosphokinase, D-dimer, troponin, creatine and urea. Parameters that proved to be significant in a continuous analysis were further analyzed in a combined and stratified way into cut-off levels. For the evaluation of CPR and neutrophil/lymphocyte ratio, tertiles of distribution of values in the studied sample were determined.
• Chest Computed Tomography (CT) findings were considered not suggestive of COVID-19 when normal or in the presence of consolidation or pleural effusion, and suggestive of COVID-19 in the presence of Ground Glass (GG) image and classified as GG < 50% and GG ≥ 50%. CT was indicated on the admission of all patients with flu-like symptoms and positive COVID, as part of the care protocol, regardless of the need for O_2_ supply.
• Disease evolution: intensive care unit admission, days of hospitalization.

An analysis of the type of oxygen support and the risk of orotracheal intubation was also performed, considering the number of days of O_2_ usage, the use of O_2_ on hospital admission, and the use of an O_2_ catheter, the use of face mask, and high-flow nasal cannula.

The study was approved by the Ethics and Research Committee of *Hospital das Clínicas* (CAAE: 30270820.3.0000.0068, approved in April 11^th^, 2020). Each patient added to the data analysis was included after registration in CAAE After receiving information and reading, all participants signed the consent form.

### Statistical analysis

The quantitative variables were expressed as mean (standard deviations) and medians (interquartile range) values, and the categorical variables were presented as absolute and relative frequencies. Poisson univariate analysis with a log link function and robust variance was performed to estimate the relative risk of O_2_ use (RR) and their respective 95% Confidence Intervals (CI). The Wald test for statistical significance (p ≤ 0.05) was used.^14^ SPSS version 20.0 (IBM SPSS Statics for Windows, version 20.0. Armonk, NY: IBM Corp) was used for data analysis.

## Results

**Fig. 1:**
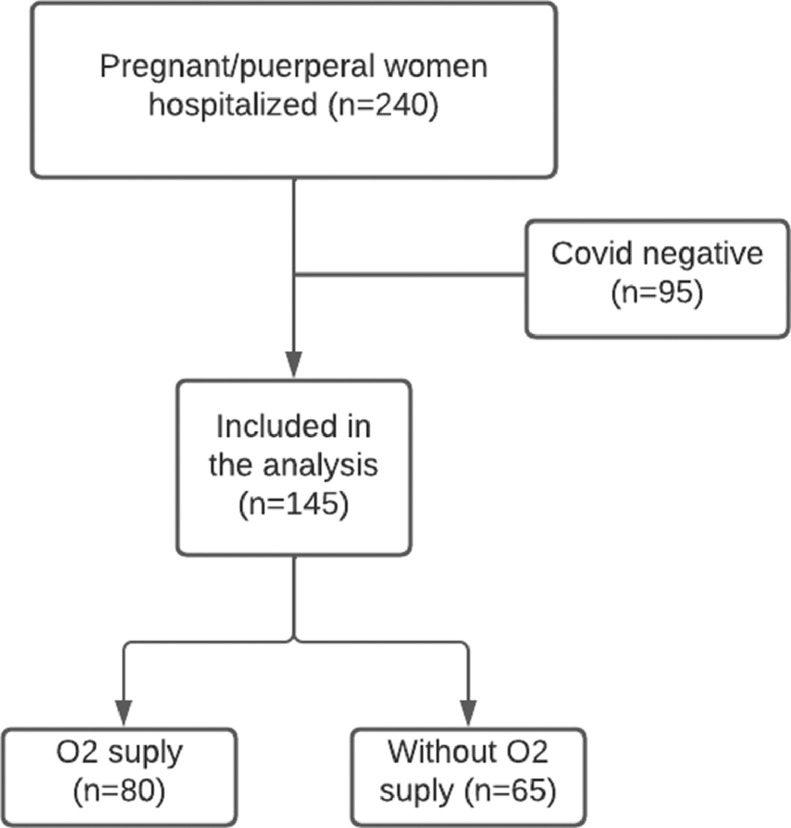
Studied population.

During the study period from April to October 2020, 240 pregnant/puerperal women with suspected COVID-19 were hospitalized. Of these, 95 tested negative for SARS-CoV-2, leaving 145 patients (144 pregnant women and 1 postpartum woman) included for analysis: 80 who used oxygen and 65 who did not (Fig. 1). The ICU admission rate was 33.1% (n = 48) and the maternal mortality rate was 4.1% (n = 6). That 80 (55.2%) patients who received oxygen during hospitalization, 41.4% (n = 60) were already hospitalized receiving O_2_, and the mean time of O_2_ use was 7.5 days (5‒15 days). The types of O_2_ supplementation used were: O_2_ catheter in 47.6% (n = 69), face mask in 29% (n = 42), high-flow nasal cannula in 11% (n = 16) and IOT in 20% (n = 29).

Clinical risk factors for the use of O_2_ were shown (Table 1): higher average maternal age (31.5 ± 6.5 vs. 27.7 ± 7.4 RR = 1.03; 95% CI 1.01‒1.05); BMI ≥ 30 (1.86; 95% CI 1.10‒3.21); smoking (1.57; 95% CI 1.16‒2.12) and chronic hypertension (1.46; 95% CI 1.09‒1.95).

**Table 1:** Comparison of demographic, clinical, and obstetrical characteristics between the COVID-19 patients who did not use O_2_ with those who used O_2_ during hospital admission.

Characteristics	O_2_ use (n = 80)	No O_2_ use (n = 65)	RR (95% CI)
Demographics			
Maternal age, years^a^	31.5 (6.5)	27.7 (7.4)	1.03 (1.01‒1.05)
Body mass index^b^	32.1 (28.71‒37.32)	28.7 (25.08‒31.23)	1.04 (1.02‒1.06)
< 25	9 (11.2)	16 (24.6)	Reference
≥ 25 < 30	18 (22.5)	23 (35.4)	1.22 (0.65–2.28)
≥30	53 (66.2)	26 (40.0)	1.86 (1.10–3.21)
Smoking habit (n = 144)	10 (12.5)	2 (3.1)	1.57 (1.16‒2.12)
Blood Type (n = 142)			
O type	32 (41.0)	29 (45.3)	0.92 (0.68‒1.25)
Other types	46 (59.0)	35 (54.7)	
Pre-pregnancy comorbidity			
Hypertension	18 (22.5)	6 (9.2)	1.46 (1.09‒1.95)
Pneumopathy	8 (10.0)	11 (16.9)	0.74 (0.43‒1.27)
Cardiopathy	5 (6.3)	3 (4.6)	1.14 (0.65‒1.99)
Diabetes	4 (5.0)	3 (4.6)	1.04 (0.54‒2.00)
Other^c^	2 (2.5)	4 (6.2)	0.59 (0.19‒1.86)
Obstetrical history			
Patient type			
Puerperal	5 (6.3)	6 (9.2)	0.81 (0.42‒1.58)
Pregnant	75 (93.8)	59 (90.8)	
Preeclampsia	6 (7.5)	4 (6.2)	1.09 (0.64‒1.86)
Gestational diabetes	21 (26.3)	13 (20)	1.16 (0.85‒1.59)

Data presented as number (%),

a mean (standard deviation) or

b median (interquartile range). RR, Relative Risk; CI, Confidential Interval.

c Other: reumathic disease, neurological disorders.

Patients whose reason for hospitalization was COVID-19 and those who were admitted for obstetric reasons received, respectively, 8.24 (95% CI 2.8‒24.29), and 3.44 (95% CI 1.05‒11.31) times more O_2_ in comparison to patients whose reason was admission for delivery (Table 2). The symptoms of COVID-19 with the highest risk of needing O_2_ were dyspnea (4.59; 95% CI 2.41‒8.75), cough (3.70; 95% CI 1.87‒7.32), fever (2.20; 95% CI 1.48‒3.27), asthenia (1.86; 95% CI 1.45‒2.37), fatigue (1.79; 95% CI 1.40‒2.30) and odynophagia (1.39; 95% CI 1.03‒1.88). Symptoms of anosmia (0.68; 95% CI 0.49‒0.94) and coryza (0.69; 0.49‒0.99) were associated with a lower need for O_2_ use. The risk of admission to the Intensive Care Unit (ICU) (Table 2) was 2.73 times higher in those who used O_2_ (57.5%×3%; 95% CI 2.07‒3.61). Two patients who did not require O_2_ were referred to the ICU: one had supraventricular tachycardia requiring drug cardioversion and the other had a hypertensive crisis refractory to the nitroglycerin use.

**Table 2:** Comparison of the reasons for hospitalization and the COVID-19 related aspects between COVID-19 patients who did not need O_2_ with those who needed O_2_ at hospital admission.

	O_2_ use (n = 80)	No O_2_ use (n = 65)	RR (95% CI)
**Reason for hospitalization**			
Hospital admission due to delivery and abortion	3 (3.8)	28 (43.1)	Reference
Hospital admission due to obstetric reasons	10 (12.5)	20 (30.8)	3.44 (1.05‒11.31)
Hospital admission due to COVID-19	67 (83.8)	17 (26.2)	8.24 (2.8‒24.29)
**COVID-19**			
Gestational age at onset of symptoms, weeks (n = 144) b	30.42 (25.14‒33.00)	33.57 (27.43‒37.71)	1.01 (1.00‒1.01)
Days of symptoms at admission (n = 143) b	8 (5‒10)	5 (4‒8)	1.02 (0.99‒1.05)
**Symptoms**			
Fever	61 (76.3)	25 (38.5)	2.20 (1.48‒3.27)
Cough	73 (91.3)	34 (52.3)	3.70 (1.87‒7.32)
Odinophagy	18 (22.5)	7 (10.8)	1.39 (1.03‒1.88)
Myalgia	44 (55.0)	26 (40.0)	1.31 (0.97‒1.76)
Asthenia	29 (36.3)	5 (7.7)	1.86 (1.45‒2.37)
Coryza	22 (27.5)	29 (44.6)	0.69 (0.49‒0.99)
Diarrhea	4 (5.0)	3 (4.6)	1.04 (0.54‒2.01)
Anosmia	28 (35.0)	36 (55.4)	0.68 (0.49‒0.94)
Dysgeusia	21 (26.3)	24 (36.9)	0.78 (0.56‒1.13)
Dyspnoea	72 (90.0)	24 (36.9)	4.59 (2.41‒8.75)
Headache	29 (36.3)	28 (43.1)	0.88 (0.64‒1.19)
Fatigue	27(33.8)	5 (7.7)	1.79 (1.40‒2.30)
**ICU admission**	46 (57.5)	2 (3.1)	2.73 (2.07‒3.61)
**Length of hospital stay, days**	9 (7‒18)	4 (3‒7)	1.01 (1.00‒1.01)

Data presented as number (%),

^a^mean (standard deviation) or

b median (interquartile range). RR, Relative Risk; CI, Confidential Interval; ICU, Intensive Care Unit.

Table 3 (clinical and tomographic parameters on hospital admission) shows that respiratory rate greater than or equal to 24 breaths per minute and O_2_ saturation less than 95% presented relative risks for O_2_ requirement of 2.55 (95% CI 1.82‒3.56) and 1.68 (95% CI 1.27‒2.20), respectively; CT findings with ground glass < 50% and ground glass ≥ 50% with risks of needing O_2_ respectively of 3.41 (95% CI 1.21‒9.60) and 5.33 (95% CI 1.92‒14.79).

**Table 3:** Comparison of clinical and chest tomography parameters at hospital admission between the COVID-19 patients who did not need O_2_ with those who needed O_2_ at hospital admission.

	O_2_ use (n = 80)	No O_2_ use (n = 65)	RR (95% CI)
**Clinical evaluation on admission**			
Heart rate^a^	96.5 (16.8)	92.1 (14.6)	1.01 (0.99‒1.02)
Respiratory rate^b^	26 (21‒32)	20 (18‒22)	1.03 (1.02‒1.05)
< 24	26 (32.9)	54 (83.1)	Reference
≥ 24	53 (67.1)	11(16.9)	2.55 (1.82‒3.56)
Systolic blood pressure^b^	117 (106‒130)	117 (110‒122)	1.00 (0.99‒1.01)
Dyastolic blood pressure^b^	71 (69‒81)	70 (66‒80)	1.00 (0.99‒1.01)
Body temperature^b^	36.4 (36‒36.5)	36 (36‒36.5)	1.28 (0.96‒1.72)
O_2_ Saturation^b^	96 (95‒98)	98 (98‒99)	0.98 (0.97‒0.99)
≥ 95	72 (90)	64 (98.5)	Reference
< 95	8 (10)	1 (1.5)	1.68 (1.27–2.20)
**Computer Tomography (n = 116)**			
Not COVID^c^	3 (3.9)	13 (32.5)	Reference
Ground Glass < 50%	48 (63.2)	27 (67.5)	3.41 (1.21‒9.60)
Ground Glass ≥ 50%	25 (32.9)	0 (0)	5.33 (1.92‒14.79)

Data presented as number (%),

a mean (standard deviation) or

b median (interquartile range). RR, Relative Risk; CI, Confidential Interval

c Not Covid, Normal, consolidation, pleural effusion.

Regarding laboratory tests (Table 4), there was a higher risk of needing oxygen for values of: hemoglobin < 11 mg/dL (1.38; 95% CI 1.04–1.82); lymphocytes < 1.50 mil/mm^3^ (1.75; 95% CI 1.11–2.75) or less than < 1.00 mil/mm^3^ (1.98; 95% CI 1.27–3.07); C-Reactive Protein (CPR) levels between 21 to 66.6 mg/L (2.28; 95% CI 1.33–3.91) and CRP > 66.6 mg/L (2.78; 95% CI 1.67–4.62). The association of CRP > 21 mg/L, hemoglobin < 11 g/d/L and Lymphocytes < 1500 mm^3^ had an RR of 4.97 (95% CI 1.74–14.14) for the O_2_ need (Table 5).

**Table 4:** Comparison of laboratorial parameters at hospital admission between the COVID-19 patients who did not need O_2_ with those who needed O_2_ during hospital admission.

Laboratorial evaluation on admission	O_2_ use (n = 80)	No O_2_ use (n = 65)	RR (95% CI)
Hemoglobin (n = 143)^b^ g/dL	11.0 (10.0‒12.0)	12.0 (10.0‒13.0)	0.88 (0.81‒0.97)
≥ 11 g/dL	46 (57.5)	47 (74.6)	Reference
< 11 g/dL	34 (42.5)	16 (25.4)	1.38 (1.04–1.82)
Leukocytes (n = 143)^b^ mil/mm^3^	8.42 (6.21‒10.82)	9.82 (6.33‒13.03)	1.00 (1.00‒1.00)
Lymphocytes (n = 143)^b^ mil/mm^3^	1.09 (0.785‒1.425)	1.36 (1.05‒1.88)	0.99 (0.99‒1.00)
≥ 1.50 mil/mm^3^	16 (20)	30 (46.2)	Reference
< 1.50 mil/mm^3^	31 (38.8)	20 (30.8)	1.75 (1.11–2.75)
< 1.00 mil/mm^3^	33 (41.2)	15 (23.1)	1.98 (1.27–3.07)
Neutrophils (n = 143)^b^ mil/mm^3^	6.870 (4.410‒9035)	7.250 (4.790‒10.670)	1.00 (1.00‒1.00)
Neutrophils/Lymphocytes (n = 143)^b^	5.9 (3.82‒9.30)	5.0 (2.93‒6.71)	1.03 (1.01‒1.05)
< 4	24 (30)	24(38.1)	Reference
≥ 4 ≤ 6.8	23 (28.7)	24(28.1)	0.98 (0.65–1.47)
> 6.8	33 (41.2)	15 (23.8)	1.38 (0.98–1.93)
Platelets (n = 143)^b^ mil/mm^3^	224 (187.5‒268)	220 (155‒275)	1.00 (1.00‒1.00)
CRP (n = 138) mg/L b	66.0 (32.0‒116.0)	18.3 (7.1‒44.0)	1.005 (1.003‒1.007)
< 21 mg/L	12 (15)	33 (50.8)	Reference
≥ 21 ≤ 66.6 mg/L	28 (35)	18 (27.7)	2.28 (1.33–3.91)
> 66.6 mg/L	40 (50)	14 (21.5)	2.78 (1.67–4.62)
AST (n = 141) U/L^b^	25 (19‒38)	19 (15‒27)	1.00 (1.00‒1.00)
ALT (n = 141) U/L^b^	18 (13‒26)	15 (10‒22)	1.00 (1.00‒1.00)
LDH (n = 129) U/L^b^	260 (197‒326)	200 (170‒251)	1.00 (1.00‒1.00)
CPK (n = 120) U/L^b^	51 (30‒97)	57 (29‒87)	1.00 (0.99‒1.00)
D Dimer (n = 132) ng/mL b	1.199 (936‒1821)	1.675 (990‒2.316)	1.00 (1.00‒1.00)
Troponin (n = 120) ng/mL b	0.005 (0.004‒0.007)	0.005 (0.004‒0.007)	1.16 (0.81‒1.65)ⱡ
Creatinine (n = 140) mg/dL^b^	0.52 (0.44‒0.61)	0.56 (0.48‒0.63)	0.64 (0.33‒1.24)
Urea (n = 141) mg/dL^b^	13 (11‒19)	16 (13‒19)	0.99 (0.97‒1.01)

Data presented as number (%)

^a^ mean (standard deviation) or

b median (interquartile range). RR, Relative Risk; CI, Confidential Interval; CRP, C-Reactive Protein; AST, Aspartate Aminotransferase; ALT, Alanine Aminotransferase; LDH, Lactic Dehydrogenase; CPK, Creatinophosphokinase. ⱡlog 10.

**Table 5:** Risk estimates for oxygen use with combined laboratory parameters.

Laboratory parameters	RR (95% CI)
CPR ≥ 21 mg/L	3.33 (1.02–10.92)
CPR ≥ 21 mg/L and Hb < 11 g/dL	3.75 (1.17–12.01)
CPR ≥ 21 mg/L and Ly < 1.5 mil/mm^3^	3.96 (1.38–11.34)
CPR ≥ 21 mg/L and Ly < 1.5 mil/mm^3^ and Hb < 11 g/dL	4.97 (1.74–14.14)

RR, Relative Risk; CI, Confidential Interval; CRP, C-Reactive Protein; Ly, Lymphocyte; Hb, Hemoglobin.

All types of O_2_ use were associated with the need for orotracheal intubation. The use of an O_2_ catheter had a RR of 2.89 (95% CI 1.37‒6.09); the use of a face mask had a RR of 6.44 (95% CI 3.09‒13.37) and the use of a high-flow nasal cannula, RR of 4.24 (95% CI 2.42‒7.45).

## Discussion

### Principal findings

The need for O_2_ in pregnant and postpartum women with COVID-19 is associated with clinical factors (advanced age, obesity, hypertension, smoking), symptoms (dyspnea, cough, fever, asthenia, fatigue, and odynophagia), physical and laboratory examination and tests of images on admission (respiratory rate ≥ to 24 breaths per minute, O_2_ saturation < 95%, ground-glass CT, hemoglobin values < 11 mg/dL, lymphocytes < 1.50 mil/mm^3^ and C-Reactive Protein [CPR] levels > 21 mg/L). Furthermore, the combination of CRP ≥ 21 mg/L with hemoglobin < 11.0 g/dL and lymphopenia < 1500 mm^3^ increased the risk of supplemental O_2_ almost fivefold. The authors studied the two most frequent obstetric pathologies, preeclampsia, and gestational diabetes, and the presence of neither pathologies was shown to be a risk factor for the use of O_2_, although pregnant women with COVID-19 hospitalized for obstetric complications are at greater risk of using oxygen than those admitted for delivery and abortion. The results also show that the risk of orotracheal intubation can also be estimated and increases as measures of oxygen supplementation progress.

### Comparison with results of previous studies

Since the first case reports of COVID-19 in non-pregnant women, overweight has been appointed as an important risk factor for clinical deterioration. Studies in pregnant women have confirmed that, as demonstrated in the present study.^9^^,^^15^, ^16^, ^17^, ^18^ It is observed that pre-existing chronic arterial hypertension was a risk factor for the use of oxygen, but few studies on pregnant women corroborate the present findings.^17^ This difference may be due to the high prevalence of chronic arterial hypertension in the studied population. Among those with pneumopathy, the authors had 19 patients in the entire sample, of which 8 (10%) needed O_2_ and 11 (16.9%) did not. Although numerically the patients with pneumopathy required less O_2_ supplementation, there was no statistically significant difference between the groups. This might have been because most of these 19 patients had only mild asthma as their underlying lung disease. Although most studies show lung disease as a risk factor for the clinical worsening of COVID-19.^15^^,^^17^^,^^18^ the present findings have already been seen by La Verde et al.,^9^ who did not find asthma as a risk factor. for aggravation of pregnant women.

The present results are in agreement with the findings of Hessami et al.,^15^ who point to older maternal age as a risk factor for clinical worsening in pregnant women. In general population studies, blood type O has already been appointed as a protective factor for the unfavorable evolution of COVID-19.^13^ However, such evidence was found neither in the sample nor in a study by Latz et al.^19^ The median gestational age at the onset of symptoms was 30,42 weeks in the group that required O_2_ and 33.57 weeks in the group that did not use O_2_, but with no statistically significant difference. Although there is consensus in the literature that uterine volume is a mechanical factor that interferes with ventilation, this was not what the authors observed in the present study.

An interesting finding of this study was that pregnant women with COVID-19 who are hospitalized for obstetric indications, even with mild symptoms, have a 3.44 times greater chance of O_2_ need than those admitted for delivery or abortion. This may suggest that the inflammatory state, present in some complications of pregnancy, may contribute to the worsening of COVID-19. It is important to note that among the patients hospitalized for delivery or abortion, none of these pregnancies was interrupted by the worsening of COVID-19.

The type of symptom that also determines the worsening of COVID-19 in pregnant women has been little studied. Savasi et al.^16^ observed that fever and dyspnea were associated with more severe clinical conditions. Furthermore, in the present study, it was also observed that cough, asthenia, and fatigue were associated with a higher risk of oxygen use. These symptoms point to a systemic involvement, while anosmia and coryza, which are symptoms more suggestive of upper airway involvement, were associated with a lower risk of oxygen use.

In the present study, increased respiratory rate and low O_2_ saturation at hospital admission were associated with a greater chance of requiring oxygen. Similar results were observed in an Italian cohort study,^16^ in which the authors also observed an increase in maternal heart rate as a risk factor, but this fact was not observed in the present series.

In agreement with studies, the following laboratory alterations were observed as a risk factors for oxygen use: decreased hemoglobin rate, lymphopenia, increased neutrophil/lymphocyte ratio, and higher levels of C-reactive protein.^20^, ^21^, ^22^, ^23^ Other predictors of clinical worsening in patients with COVID-19 in non-pregnant population cohorts, such as increased DHL, increased D-dimer, and increased creatinine, were not observed in the present study.^20^, ^21^, ^22^, ^23^, ^24^, ^25^

As in non-pregnant women, the presence of ground glass findings on chest CT is a relevant predictor of the need to use O_2_, and the risk increases according to the percentage of involvement of the lung parenchyma.^26^

All pregnant women who required O_2_ supplementation had a higher risk of orotracheal intubation. The simple use of an O_2_ catheter implies an approximately three times greater risk of orotracheal intubation, demonstrating the need for greater surveillance of these patients.

### Clinical implications

It is known that pregnant women are at higher risk for severe COVID-19 compared to the general population, especially with regard to admission to intensive unit care and the need for orotracheal intubation.^1^ However, it is difficult to identify pregnant and postpartum women who will develop a severe respiratory conditions and, consequently, will need O_2_. Brazil is currently facing an increase in maternal mortality from COVID-19, which may be associated with the increase in the number of cases, but also with the lack of access to the health system by pregnant and postpartum women. It is observed that of the pregnant and postpartum women who died because of COVID-19 in Brazil, one in five was not admitted to the intensive unit care and one in three did not have access to the orotracheal intubation.^4^ The risk factors for oxygen supplementation found in this study can be extremely important to identifying the group of pregnant and postpartum women with a higher risk of needing O_2_ and, consequently, a greater chance of being admitted to intensive unit care or mechanical ventilation. This can reduce maternal mortality, both in Brazil and in those who observed an increase in maternal mortality due to COVID-19.

### Strengths and limitations

The strength of this study is the access to clinical, laboratory, and history data of a relevant number of pregnant and postpartum women with COVID-19, admitted to a single hospital, followed by the same protocol, a fact not observed in other studies.^9^^,^^10^^,^^15^, ^16^, ^17^, ^18^ As a limitation of the study, a considerable percentage of pregnant women were admitted while already receiving O_2_, making it impossible to obtain a predictive model of O_2_ requirement, though not invalidating the proposed analysis of risk factors.

Another limitation of the study was that sociodemographic characteristics such as income, education, and ethnicity, which are correlated with causes of higher risk of contamination and worse outcomes,^4^, ^5^, ^6^ were not collected at the time of patient inclusion.

## Conclusions

In pregnant women, a population at higher risk for developing critical forms of COVID-19, BMI ≥ 30, smoking, chronic hypertension, obstetric reasons for hospitalization, respiratory rate ≥ 24 cycles/min, O_2_ saturation < 95%, ground glass on CT and combination of altered laboratory parameters were identified as risk factors for oxygen need. These findings help to define the population with the greatest chance of clinical deterioration and who need access to more resources in health care systems.

## Authors' contributions

Conceptualization: F.S.B., M.L.B. and R.P.V.F; Data collect: F.S.B., C.F.P., U.T.G. and HC-FMUSP-Obstetric COVID-19 Study Group; Formal analysis: F.S.B., S.V.P. and R.P.V.F.; Methodology: F.S.B., M.L.B. and R.P.V.F; Supervision: R.P.V.F.; Writing ‒ original draft: F.S.B.; Writing ‒ review & editing: L.M.M., M.L.B. and R.P.V.F. All authors have read and agreed to the published version of the manuscript.

## Funding

CAPES (88881.504727/2020-01) and HCComvida (02.25). The funders had no role in the study design, data collection and analysis, decision to publish, or preparation of the manuscript.

## Ethical approval

The study was approved by the Ethics and Research Committee of Hospital das Clínicas (CAAE: 30270820.3.0000.0068).

## Informed consent

All the patients signed informed consent.

## Declaration of Competing Interest

The authors declare no conflicts of interest.

## References

[1] Zambrano L.D., Ellington S., Strid P., Galang R.R., Oduyebo T., Tong V.T. (2020). Update: characteristics of symptomatic women of reproductive age with laboratory-confirmed SARS-CoV-2 infection by pregnancy status ‒ United States, January 22 – October 3, 2020. MMWR Morb Mortal Wkly Rep.

[2] Martinez-Portilla R.J., Sotiriadis A., Chatzakis C., Torres-Torres J., Espino y Sosa S., Sandoval-Mandujano K. (2021). Pregnant women with SARS-CoV-2 infection are at higher risk of death and pneumonia: propensity score matched analysis of a nationwide prospective cohort (COV19Mx). Ultrasound Obstet Gynecol.

[3] Qeadan F., Mensah N.A., Tingey B., Stanford J.B. (2021). The risk of clinical complications and death among pregnant women with COVID-19 in the Cerner COVID-19 cohort: a retrospective analysis. BMC Pregnancy Childbirth.

[4] Francisco R.P.V., Lacerda L.R.A.S. (2021). Obstetric Observatory Brazil ‒ COVID-19: 1031 maternal deaths because of COVID-19 and the unequal access to health care services. Clinics.

[5] Joseph N.T., Wylie B.J. (2020). Maternal deaths in Brazil from severe COVID-19 respiratory disease: time for a global commitment to ending health disparities. BJOG.

[6] Nakamura-Pereira M., Knobel R., Menezes M.O., Andreucci C.B., Takemoto MLS. (2021). The impact of the COVID-19 pandemic on maternal mortality in Brazil: 523 maternal deaths by acute respiratory distress syndrome potentially associated with SARS-CoV-2. Int J Gynaecol Obstet.

[7] Yap M., Debenham L., Kew T., Chatterjee S.R., Allotey J., Stallings E. (2020). Clinical manifestations, prevalence, risk factors, outcomes, transmission, diagnosis, and treatment of COVID-19 in pregnancy and postpartum: a living systematic review protocol. BMJ Open.

[8] Heldt F.S., Vizcaychipi M.P., Peacock S., Cinelli M., McLachlan L., Andreotti F. (2021). Early risk assessment for COVID-19 patients from emergency department data using machine learning. Sci Rep.

[9] la Verde M., Riemma G., Torella M., Cianci S., Savoia F., Licciardi F. (2021). Maternal death related to COVID-19: A systematic review and meta-analysis focused on maternal co-morbidities and clinical characteristics. Int J Gynaecol Obstet.

[10] DeBolt C.A., Bianco A., Limaye M.A., Silverstein J., Penfield C.A., Roman A.S. (2021). Pregnant women with severe or critical coronavirus disease 2019 have increased composite morbidity compared with nonpregnant matched controls. Am J Obstet Gynecol.

[11] Manual de recomendações para a assistência à gestante e puérpera frente à pandemia de Covid-19 [recurso eletrônico] /Ministério da Saúde, Secretaria de Atenção Primária à Saúde, Departamento de Ações Programáticas e Estratégicas. –2. ed. – Brasília: Ministério da Saúde, 2021. Available from: http://bvsms.saude.gov.br/bvs/publicacoes/manual_assistencia_gestante_puerpera_covid-19_2ed.pdf ISBN 978-65-5993-074-6.

[12] Pacheco L.D., Saad A.F., Saade G. (2020). Early acute respiratory support for pregnant patients with coronavirus disease 2019 (COVID-19) Infection. Obstet Gynecol.

[13] Zhang Y., Garner R., Salehi S., la Rocca M., Duncan D. (2021). Association between ABO blood types and coronavirus disease 2019 (COVID-19), genetic associations, and underlying molecular mechanisms: a literature review of 23 studies. Ann Hematol.

[14] Altman E., Mounir I., Najid F.Z., Perlaza S.M. (2020). On the true number of COVID-19 infections: Effect of sensitivity, specificity and number of tests on prevalence ratio estimation. Int J Environ Res Public Health.

[15] Hessami K., Homayoon N., Hashemi A., Vafaei H., Kasraeian M., Asadi N. (2020). COVID-19 and maternal, fetal and neonatal mortality: a systematic review. J Matern Fetal Neonatal Med.

[16] Savasi V.M., Parisi F., Patanè L., Ferrazzi E., Frigerio L., Pellegrino A. (2020). Clinical findings and disease severity in hospitalized pregnant women with coronavirus disease 2019 (COVID-19). Obstet Gynecol.

[17] Lokken E.M., Walker C.L., Delaney S., Kachikis A., Kretzer N.M., Erickson A. (2020). Clinical characteristics of 46 pregnant women with a severe acute respiratory syndrome coronavirus 2 infection in Washington State. Am J Obstet Gynecol.

[18] Pierce-Williams R.A.M., Burd J., Felder L., Khoury R., Bernstein P.S., Avila K. (2020). Clinical course of severe and critical coronavirus disease 2019 in hospitalized pregnancies: a United States cohort study. Am J Obstet Gynecol MFM.

[19] Latz C.A., DeCarlo C., Boitano L., Png C.Y.M., Patell R., Conrad M.F. (2020). Blood type and outcomes in patients with COVID-19. Ann Hematol.

[20] Pan F., Yang L., Li Y., Liang B., Li L., Ye T. (2020). Factors associated with death outcome in patients with severe coronavirus disease-19 (Covid-19): A case-control study. Int J Med Sci.

[21] Feng X., Li S., Sun Q., Zhu J., Chen B., Xiong M. (2020). Immune-inflammatory parameters in COVID-19 cases: A systematic review and meta-analysis. Front Med (Lausanne).

[22] Wang H., Zhang Y., Mo P., Liu J., Wang H., Wang F. (2020). Neutrophil to CD4+ lymphocyte ratio as a potential biomarker in predicting virus negative conversion time in COVID-19. Int Immunopharmacol.

[23] Fu J., Kong J., Wang W., Wu M., Yao L., Wang Z. (2020). The clinical implication of dynamic neutrophil to lymphocyte ratio and D-dimer in COVID-19: A retrospective study in Suzhou China. Thromb Res.

[24] Huang I., Pranata R., Lim M.A., Oehadian A., Alisjahbana B. (2020). C-reactive protein, procalcitonin, D-dimer, and ferritin in severe coronavirus disease-2019: a meta-analysis. Ther Adv Respir Dis.

[25] Liu F., Li L., Xu M .D.da, Wu J., Luo D., Zhu Y.S. (2020). Prognostic value of interleukin-6, C-reactive protein, and procalcitonin in patients with COVID-19. J Clin Virol.

[26] San-Juan R., Barbero P., Fernández-Ruiz M., López-Medrano F., Lizasoáin M., Hernández-Jiménez P. (2020). Incidence and clinical profiles of COVID-19 pneumonia in pregnant women: a single-centre cohort study from Spain. EClinicalMedicine.

